# Design, simulation and analysis of micro electro‐mechanical system microneedle for micropump in drug delivery systems

**DOI:** 10.1049/nbt2.12013

**Published:** 2021-03-30

**Authors:** Srinivasa R. Karumuri, Hamza Mohammed, Koushik Guha, Ashok K. Puli, Ameen Einsanwi, Girija S. Kondavitee

**Affiliations:** ^1^ MEMS Research Center Department of Electronics Communication Engineering Koneru Lakshmaiah Education Foundation (Deemed to be University) Guntur Andhra Pradesh India; ^2^ National MEMS Design Center Department of Electronics Communication Engineering National Institute of Technology Silchar Assam India; ^3^ Mechanical Engineering American University of Iraq Sulaimani Iraq

## Abstract

This article reports on the mechanical strength analysis and flow characteristics of square tip and circular tip microneedles by employing highly potent drugs that are given in extremely little quantity (microlitres) using MEMS technology, which proves to be a significant component of micropump in the application of Bio‐MEMS. These microneedles are well suitable for a MEMS‐based micropump in the drug delivery systems. It is an essential part of the micropump through which the drug is released into the patient’s body. The proposed microneedles can withstand a stress of 23 MPa and 20 KPa. An extensive investigation on selection of material for the microneedle is carried out to meet the requirements of the biocompatibility and high yield, as well as tensile strength. As mighty drugs such as vasopressin, atropine and digoxin are administered in large quantities, the microneedle is designed so as to deliver 800 µl of drug, with each microneedle delivering 90 µl. in a 3 3 array. 3 × 3 array releasing 90 µl.

## 1 INTRODUCTION

Microneedle is a striking component of any drug delivery system as it is the medium for ultimate release of drug into the patient’s body. The release of drug is accompanied with the needle piercing into the skin that is considered uncomfortable causing soreness especially in the case of infants and old people. In the absence of secreted hormones in certain medical conditions, there is a large need for its compensation to forbid the fatal conditions. Therefore, highly potent drugs are given to patients in small quantities within the prescribed time. The quantity of such drugs varies from 0.5  to 0.8 ml, which is upto 800 µl. For the painless and efficient drug delivery in vulnerable medical conditions, MEMS technology can be utilised in the design of biocompatible microneedles that pierce into skin painlessly and release the drug efficiently [1].

The design of MEMS microneedle is done in accordance with the ways and point of delivery. The drug can be delivered intravenously, intramuscularly and subcutaneously. In subcutaneous delivery, the needle is pierced into the tissue that is just below the skin, after which, the drug carried away in the blood. In case of intramuscular injection, the drug should be injected into the muscle. This criterion is chosen for abundant drug delivery cases. It also requires a long microneedle to penetrate into muscle passing through skin and fatty tissues. In this case, the absorption of the drug depends on the blood supply to the muscle. The sparser the blood supply, the longer it takes for the drug to be absorbed. For the intravenous delivery, the microneedle is inserted into the vein. The drug can be given instantaneously or can be infused continuously. The best method of injection is the intravenous, as it accommodates a well‐controlled manner of drug release for the prescribed dosage without causing pain and damage to the tissues although its insertion is typical. The effect of drug on the body can be seen immediately when injected intravenously. An array of MEMS microneedle is suitable for the delivery of minute quantity and highly potent drugs, which enable the painless release of drug [2].

A microneedle with a cylindrical body accompanied with side openings is designed and fabricated by employing BI mask technique that enables sharp tips. Its characteristics of high density, robustness and side openings cease the clogging during insertion [3]. The mechanism involving in the insertion process of microneedle into the skin is discussed using the Galerkin technique. Hertzian theory and Love's thin rod theory are used in the analysis of interfacial contact stress and stiffness coefficient, respectively. The analysis is carried out at pre‐ and post‐puncture of the microneedle into the tissue under a frictionless condition [4]. Hollow microneedle has been designed and fabricated using direct laser writing. Its maximum bending force is 0.022 N, and compressive force is 0.27 N with a flow rate of 0.93 µl/s [5]. A work on the centre to centre spacing of the microneedles was performed to analyse the effect of microneedle penetration into the skin. The penetration varied from 5% to 44% at a base diameter of 300 µm and 9% to 64% at 400 µm diameter [6]. An array of 16 microneedles with a tip diameter of 75 µm is designed for the delivery of insulin. It was observed that the blood glucose level fell by 47% for 4 h of delivery [7]. Fabrication of silicon hollow microneedles is presented for cardiovascular disorder. It is noted that the skin piercing pressure is 3.18 MPa at a maximum stress of 3.26 MPa. Through coupled multified analysis, the proposed micropump integrated microneedle device shows proportional increase in deflection with applied voltage but is inversely proportional with frequency, which is varied from 20H to 250H [8]. Another work on the optimisation of square microneedle array was designed. The pressure was noted as 0.089 MP [9]. Solid microneedles made of silicon were fabricated using Tetramethylammonium hydroxide (TMAH) etching factors with higher aspect ratio at a of temperature of 90°. It was observed that the etching became uncontrollable and rapid when the temperature was increased beyond 90° [10].

The article is presented under the following sections a Section 2 explains the structure, mechanism and design considerations of the microneedle. Section 3 provides the results and discussions on the flow characteristics and mechanical strength of the proposed microneedle followed by Conclusion in Section 4.

## 2 DRUG DELIVERY SYSTEM: THE STRUCTURE AND ITS MECHANISM

**FIGURE 1:**
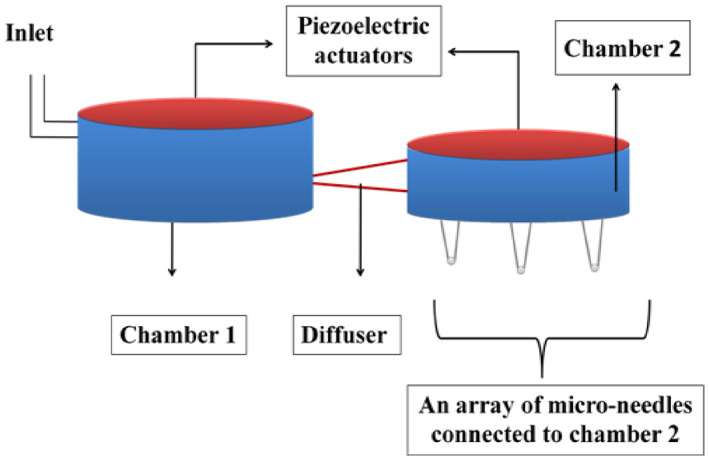
The structure of micro‐pump for drug delivery system

The micropump employed for drug delivery consists of three main components, a piezoelectric actuator for creating pressure in the chamber, a diffuser, which connects channel to both the chambers and an array of microneedles for the ultimate delivery of drug into the patient’s body. The micropump has two chambers for the storage of drug with piezoelectric actuators laid over those. Chambers one and two are connected through a diffuser that acts like a channel for the flow of drug. Chamber two is connected to an array of microneedles that ultimately deliver the drug. Figure 1 shows the design of the micropump.

As a result, the inner layer of the piezoelectric actuator deflects downwards. This creates pressure in chamber one, which is filled with the drug, and thereby, the drug escapes into chamber two through the diffuser. Now, chamber two gets filled with the drug. Again, electric potential is applied to the actuator of chamber two. Due to the pressure created, the drug is forced into the array of microneedles. The pressure created is sufficient for the microneedle to penetrate through the skin and release the drug into the patient’s body.

### 2.1 Microneedle

Microneedles are categorised into four groups, namely, solid microneedle, hollow microneedle, drug‐coated microneedle and dissolving microneedle. Solid microneedles help in reducing the permeability of the skin before the drug is delivered. These can be coated with water soluble drugs that dissolve under the skin. Otherwise, microneedles can be made of polymers that can dissolve in the skin after insertion. The hollow microneedles are similar to conduits that deliver the drug into the tissue [11]. Microneedles can be defined as applicators that are used to deliver drugs or vaccines. Microneedles can be made with various materials like silicon, titanium, stainless steel and polymers, but bio compatibility of the material must be assured. Some microneedles can be made with the drug that needs to be delivered, that is, they are shaped in the form of a needle to ensure penetration through the skin. Microneedles can vary in every aspect such as size and shape. The outstanding feature of such microneedle arrays is the painless penetration into the skin [12]. Microneedles facilitate the administration of large molecules of the drug painlessly. They provide faster healing at the site of insertion compared to a needle. They enables the prevention of microbial penetration. The duration of erythema is less as they show good tolerability and also they ensure the rapid delivery of drug, thus shielding the collapse of veins due to repetitive insertion [13].

### 2.2 The structure of the microneedle

**FIGURE 2:**
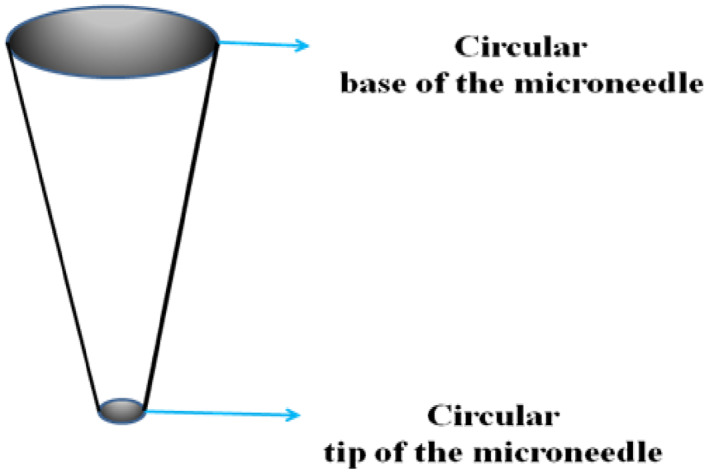
The outline of a circular tip microneedle

**FIGURE 3:**
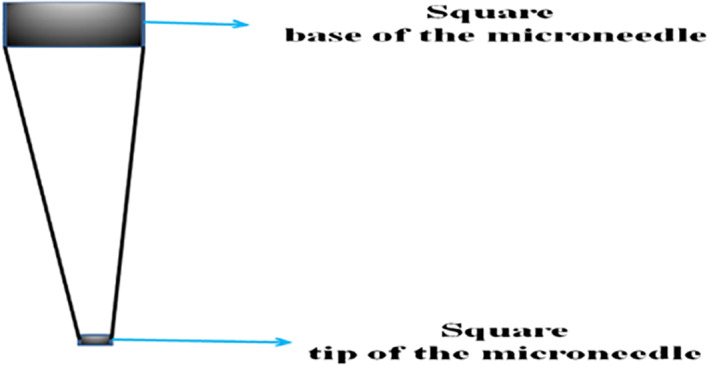
The outline of a square tip microneedle

The microneedle is designed based upon the quantity of drug that needs to be delivered. Thereby, the capacity and volume of the microneedle array are defined. The tip of the microneedle must be sharp enough to penetrate through the skin. It must also have strength to oppose the hard surface of skin. Therefore, the tip of the microneedle can be circular, square, pentagonal, hexagonal or triangular in shape. In this article, square and circular shapes are considered as shown in Figures 2 and 3. Highly potent drugs such as vasopressin, atropine and digoxin are considered are usually given in micro litre quantities. The dimensions of the microneedle are fixed to accommodate 0.8 ml of drug, which is equal to 800 µl. An array of microneedle is considered, which aims to deliver 800–900 µl of drug, with each microneedle releasing 89 µl of drug into the skin. The height of the microneedle with the circular tip and the square tip is 5460 and 4500 µm, respectively.

### 2.3 Material selection

Material selection is a significant criterion in the design of a microneedle, which is part of the micropump in the drug delivery system. The selection of the material must be made appropriately, which is biocompatible, and posses an yield strength which is less than the bending force of the microneedle to prevent the breakage of microneedle before and after insertion. The biomaterials are the key ingredients in the bio medical devices. Different groups of materials such as polymers, ceramics, composites and metals are used in various applications of medical devices such as dentistry, pace makers, implants, reservoirs and micro pumps. Before considering the material that can be used for building a biomedical device, such as the micropump, the adjustability of the material with the site of delivery into the patient’s body must be checked. Organs such as ligaments, tooth and bones are made up of hard tissues, and they possess higher tensile strength and elastic modulus. Therefore, metals are considered for these sites of operation. Polymers are considered for soft tissue applications [14].

Factors such as skin permeability, Young's modulus, tensile strength and geometry of the microneedle need to be considered. Materials with high Young's modulus and tensile strength must be selected to hinder the breakage of the microneedle. Yield strength is the point witnessed after the material begins to deform plastically. Tensile strength is the maximum stress the material can withstand. Thus, tensile strength must be greater than the yield strength of the material. Polymethyl methacrylate (PMMA) and polydimethylsiloxane (PDMS) are frequently used polymers in the making of microneedles. These polymer materials, which are biocompatible and also possess robust mechanical properties, are tested in terms of mechanical stability and safety. Simulations are carried out by employing PDMS and PMMA materials. The properties of these materials are expressed in Table 1. They have properties such as favourable thermal stability, thermal conductivity, flexibility, chemical and biological compatibility. It also provides stiffness and high mechanical firmity. These characteristics are essential in the making and efficient working of the microneedles [15, 16, 17].

**TABLE 1:** Mechanical properties of PDMS and PMMA

Material	Young's modulus(MPa)	Tensile Strength(MPa)	Yield Strength(MPa)
PMMA	2450	48‐72	4.9
PDMS	0.36–0.87	2.24	0.00174

Abbreviations: PDMA, Poly(2,5‐dimethoxyaniline); PDMS, Polydimethylsiloxane; PMMA, Polymethyl methacrylate.

### 2.4 Mathematical equations

For the efficacious working of the microneedle, the axial force acting on the microneedle must be greater than the resistance offered by the skin. The tip of the microneedle must be sharp enough to lessen the buckling effect. The radius of the microneedle tip must be smaller for the insertion force to be less [18]. The resistance of the skin at its upper layer, stratum corneum is more than in the other layers or the tissues. Hence, the opposing force from the skin reduces once the microneedle pierces the skin. The acting against the microneedle from the skin is given in Equation (1).

(1) $$F_{\text{Skin}}=P.A$$

where *P* is the pressure required to penetrate the skin by the microneedle and *A* is the area of the microneedle.

If the skin resistance is higher, then there is a probability of breakage of the microneedle followed by bending of the microneedle at the point of insertion. The bending force depends upon the yield strength of the material chosen for the microneedle, moment of inertia and the distance from the vertical axis to the outer edge. The bending force acting on the microneedle before it breaks may result in either deformation or breakage of the microneedle, and is mathematically expressed in Equation (2) [19, 20].

(2) $$F_{deformation}=\frac{2\sigma_{Y}I}{D.L}$$

where $\sigma_{y}$ is the yield strength of the material, *I* is the moment of inertia, *D* is distance from vertical axis to outer edge and *L* is the length of the microneedle.

Buckling of the microneedle can also occur, which can be defined similar to the bending force, but the difference arises due to the action of compressive axial stress. Force acting on the microneedle axially is the product of yield strength and area of the microneedle given in Equation (3), which also leads to structural deflection in the microneedle. It mainly depends on the Young's modulus of the material along with the moment of inertia, length and diameter of the microneedle as represented in Equation (4).

(3) $$F_{\text{axial}}=\sigma_{y}.A$$

(4) $$F_{\text{buckling}}=\frac{\pi^{2}EI}{L^{2}}$$

where *E* is the Young's modulus of the material, *I* is the moment of inertia, and *L* is the length of the microneedle.

## 3 RESULTS AND DISCUSSIONS

### 3.1 Flow characteristics

**FIGURE 4:**
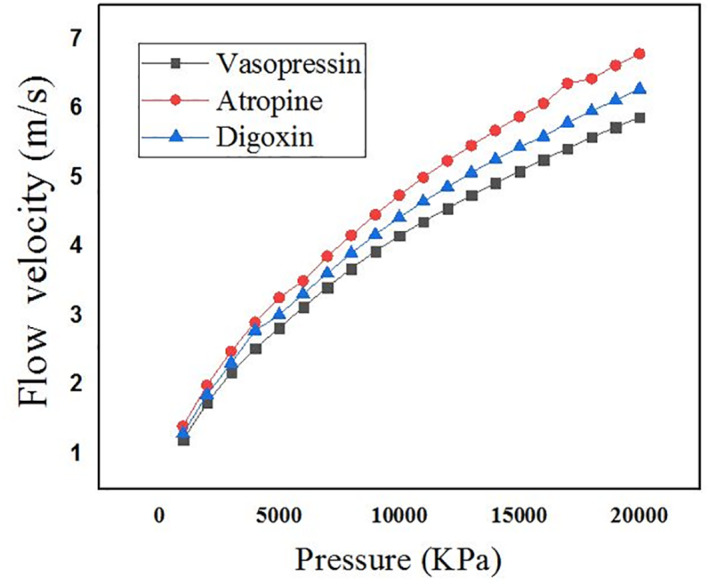
Plot showing the comparison of flow velocity for various drugs through the circular tip of microneedle

**FIGURE 5:**
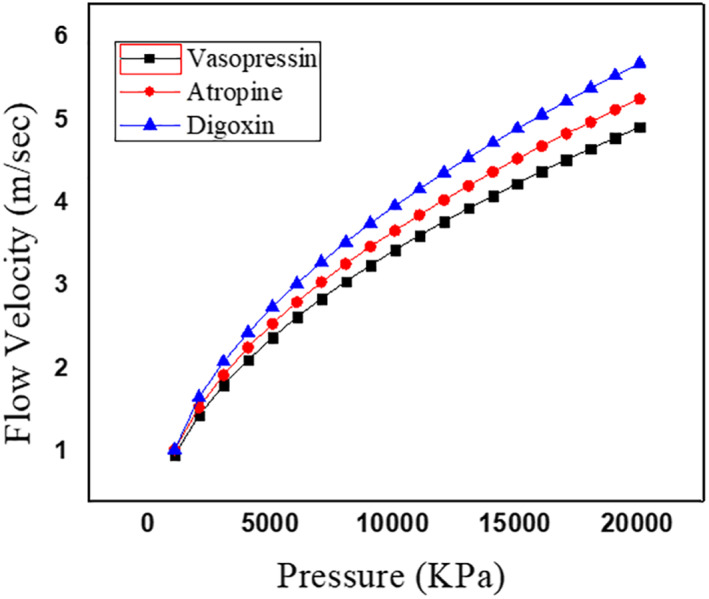
Plot showing the comparison of flow velocity for various drugs through the square tip of microneedle

**FIGURE 6:**
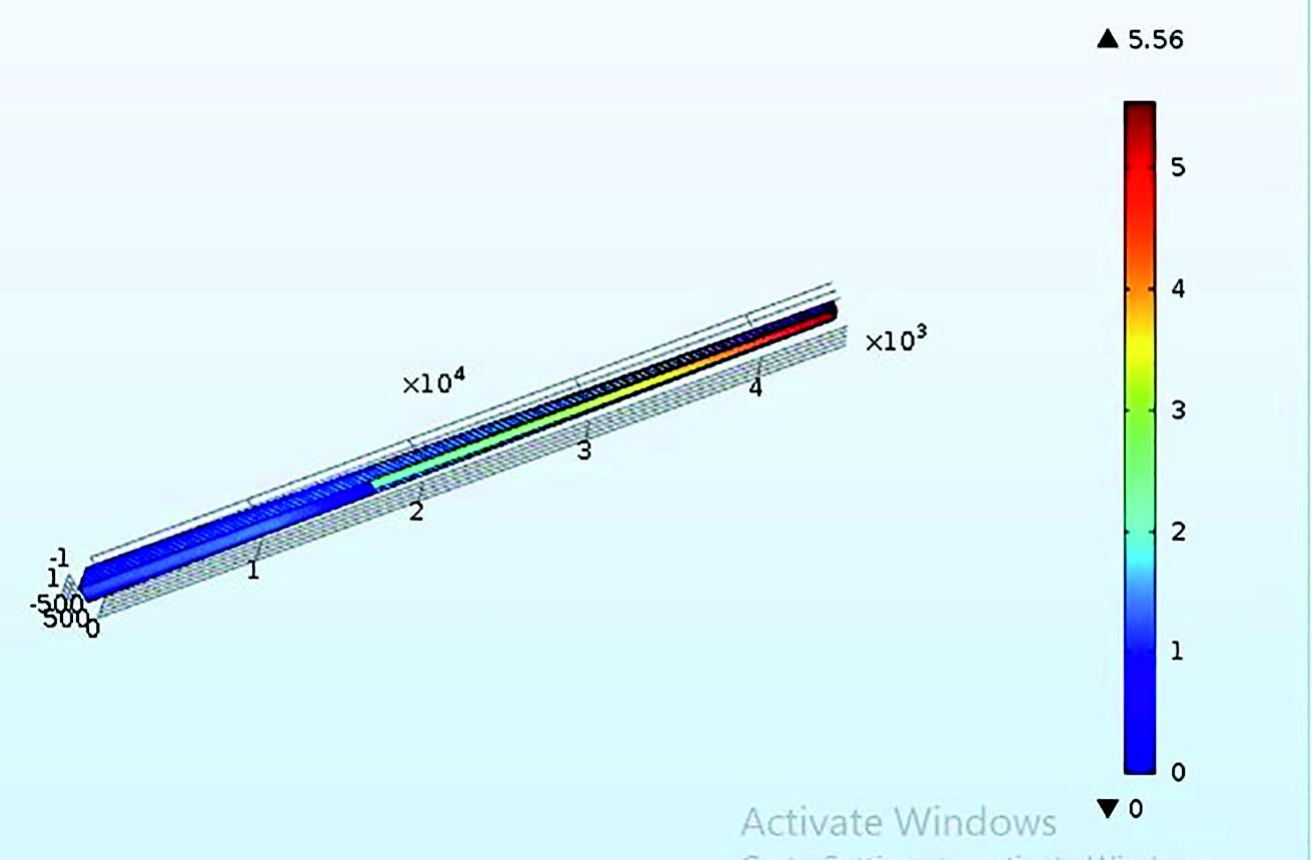
Illustration of flow through the square tip of microneedle

**FIGURE 7:**
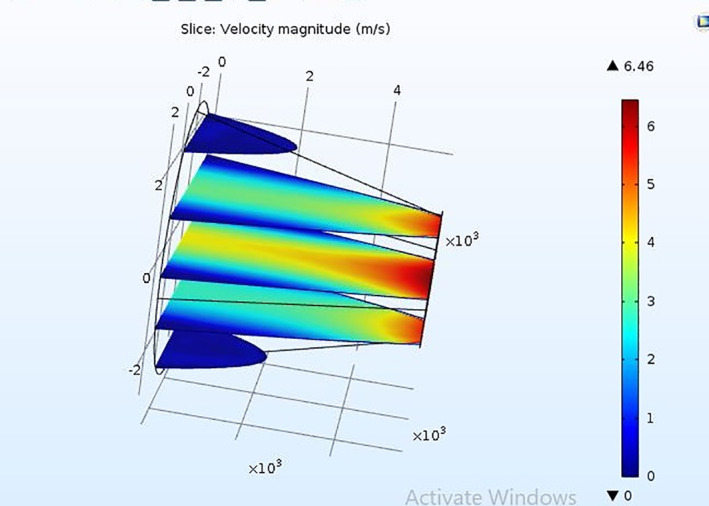
Illustration of flow through the circular tip of microneedle

The Microneedle is designed in the form of a square pyramid and cone. The base and tip of the microneedle are in circular and square shape. The concept of hollow microneedle is adopted in the work. The microneedle is designed in COMSOL MULTIPHYSICS. The microneedle is targeted to deliver 900 µl of highly potent drugs like vasopressin, atropine and digoxin. A 3 × 3 array of microneedles is considered, with each microneedle capable of releasing 90 µl of the drug into the patient’s body. As per the proposed design of the micro pump, the input to the microneedles is not force but pressure generated due to the application of voltage on the upper surface of the piezoelectric actuator [21]. The corresponding pressure at which the piezoelectric actuator deflects was observed to be in the range of 1 MPa–1 GPa before it undergoes breakage [22]. Therefore, pressure created in chamber two, where an array of microneedles is connected, must be sufficient to accompany the penetration of the microneedles into the skin. Therefore, a range of pressure varying from 1 MPa to few gigapascal is applied, and the corresponding flow velocity is observed for 90 µl of drug to get filled in each microneedle and ultimately release it into the patient’s body. The microneedle with circular and square shapes at both the ends is designed and simulated in the environment of the laminar flow in the FEM tool to estimate the flow velocity in the proposed microneedle for the aforementioned real time drugs. It is observed that in order to make the microneedles penetrate through the skin, the average flow velocity of the drugs in the case of circular tip microneedle at a pressure of 6 KPa, is 5, 6, 7 µm/s for vasopressin, atropine and digoxin, respectively. Figures 4 and 5 depict the flow velocity of drugs for circular tip and square tip microneedle respectively. Figures 6 and 7 show the simulation images of the microneedles with square tip and circular tip respectively.

### 3.2 Stress analysis

**FIGURE 8:**
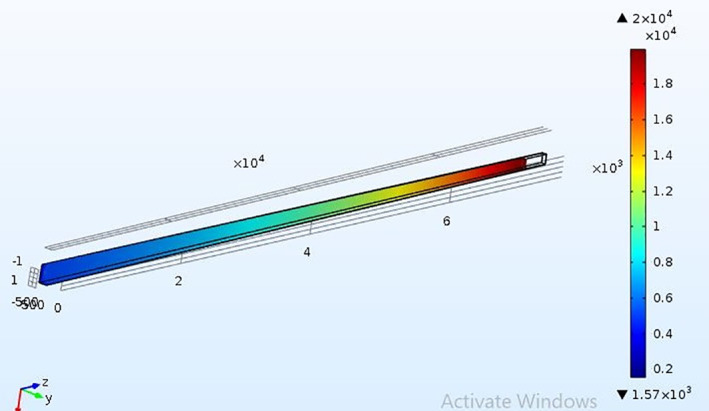
Illustration of stress through the square tip of microneedle

**FIGURE 9:**
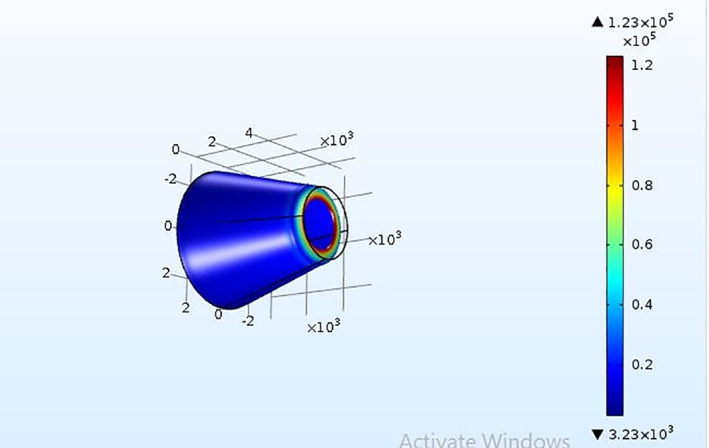
Illustration of stress through the circular tip of microneedle

**FIGURE 10:**
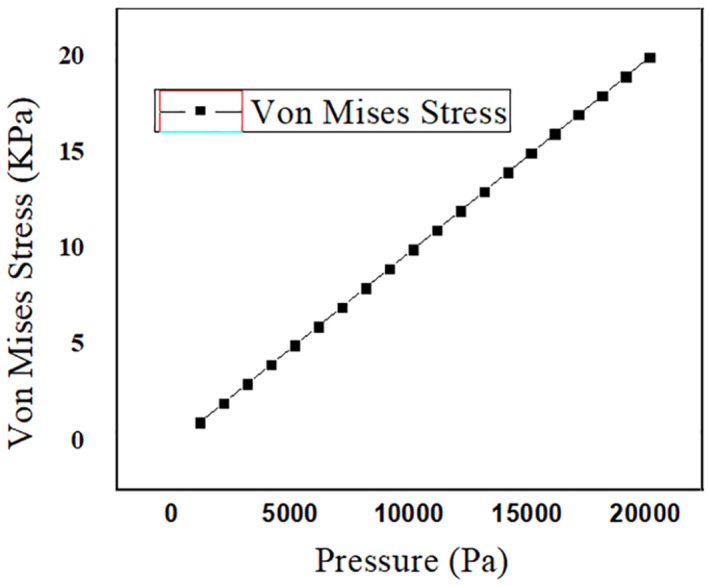
Plot showing the linear response of the circular tip microneedle to stress for the given pressure

**FIGURE 11:**
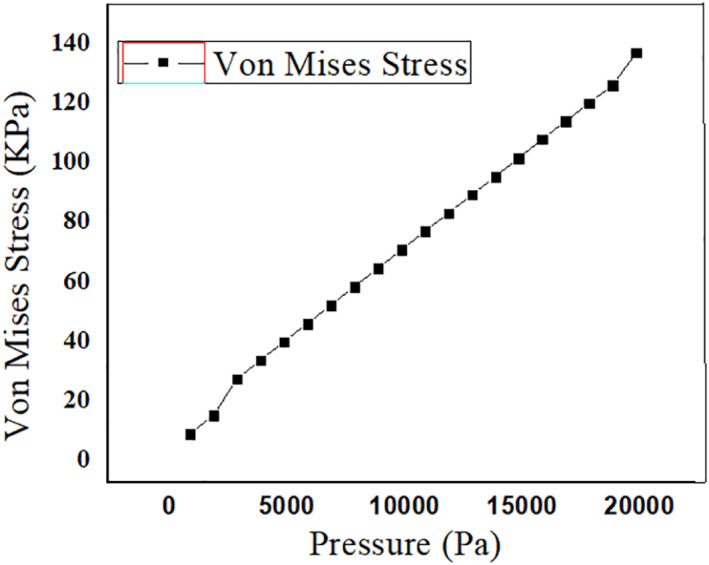
Plot showing the linear response of the square tip microneedle to stress for the given pressure

Analysis on the stress that falls on the PDMS microneedle while insertion into the skin is essential to prevent the breakage of the microneedle due to skin resistance, axial force and buckling force. Bending force is the combination of tension and compression. This force falls along the length of the microneedle from the point of its fixation. In case of excessive force, the microneedle may bend and tend to break. Buckling force is a consequence of the bending force acting on the microneedle. It is caused due to the compressive axial stress ultimately leading to the structural failure in the form of instability and bending sideways, which threatens the efficient release of the drug. Another significant criteria is the resistance offered by the skin during the puncturing of the skin by the microneedles. The microneedle must be capable of opposing the resistance and pierce into the skin [22]. The bending force given in Equation (2) must be less than the yield strength of the material chosen in the design of the microneedle. Yield strength is different from tensile strength. Tensile strength is the maximum stress a material can endure before breaking, whereas the yield strength is the stress at which the material undergoes permanent deformation and fails to work. The microneedles are simulated in the solid mechanics of COMSOL MULTIPHYSICS tool to find the breaking point of microneedle. It is a software simulation tool that supports finite element analysis. Meshing for the two geometries of the microneedles is performed for accurate results. Figures 8 and 9 show the stress acting on the square tip and circular tip microneedle. The pressure at which the microneedle can no more penetrate and break is determined for the given quantity and drugs, thereby, estimating the safety factor of the microneedles. As the tensile strength of the PMMA and PDMS microneedle is 62  and 2.24 MPa respectively, the corresponding maximum pressure is 23 MPa and 20 KPa for circular‐ and square‐shaped base and tip of the microneedles. Figures 10 and 11 depict the linear stress characteristics for both circular and square tip microneedles, respectively. It is also observed that a microneedle with four sided base and tip is less efficient compared to the one with a circular tip. Therefore, it can be drawn that the circular tip is more efficient than the polygonal surfaces in terms of holding the pressure and skin resistance in the prevention of microneedle breakage.

**FIGURE 12:**
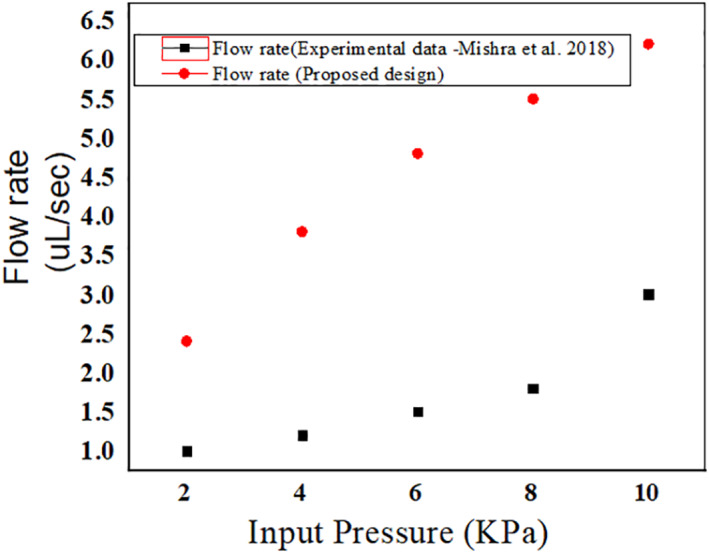
Plot showing the comparison of flow rate with the experimental flow rate obtained by Mishra et al. (2018)

An average flow rate of 4 µL/s is obtained in the proposed microneedle. The obtained data for the proposed microneedle is compared with the experimental flow rate obtained by Mishra et al. (2018). In that work, the micro needles were fabricated using SU‐8 material and direct laser writing technology. The simulated values of the flow rate in the proposed design are higher than the experimental values obtained for the fabricated microneedles as depicted in Figure 12. Also, the proposed microneedles are designed especially for a target delivery of 0.5–0.9 ml of the drug. The flow rate is observed as 6 µl/s in the proposed one, and in the other work, it is limited to 3 µl/s.

## 4 CONCLUSIONS

In this article, microneedles with square and circular base‐tip are designed and simulated in the laminar flow and solid mechanics environment of the COMSOL Multiphysics tool. The flow velocity for real time and highly potent drugs such as vasopressin, atropine and digoxin is observed by injecting the microneedle into the patient's body. The average velocity of the microneedle is noted as 5, 6 and 7 µm/s for vasopressin, atropine and digoxin, respectively, in the case of circular tip microneedle. In the event of square tip microneedles, the average velocity is 5, 5.5 and 5.9 µm/s for the aforementioned drugs, respectively. The average flow rate is observed to be 4 µl/s. The mechanical strength of the microneedle is examined by analysing the force acting on and against the microneedles made up of PDMS and PMMA. The average force acting on the microneedle is 2.0174 N. In the design, the two biocompatible polymer materials are used, PMMA is found to be the better choice as it is observed to bear more stress with its inbuilt feature of high tensile and yield strength. It is observed that the circular tip microneedles made up of PMMA gives better performance both in terms of mechanical strength and flow velocity. An array of 3 × 3 microneedles is capable of releasing 0.5–0.8 ml (≈900 µl) of highly potent drugs.
